# Supplementary material for: HiFi metagenomic sequencing enables assembly of accurate and complete genomes from human gut microbiota
Source: Nat Commun. 2022 Oct 26;13:6367. doi: 10.1038/s41467-022-34149-0 (PMC9606305; doi:10.1038/s41467-022-34149-0)
Supplement: Supplementary file 1 — Supplementary Information [file 41467_2022_34149_MOESM1_ESM.pdf]

Supplementary Figures

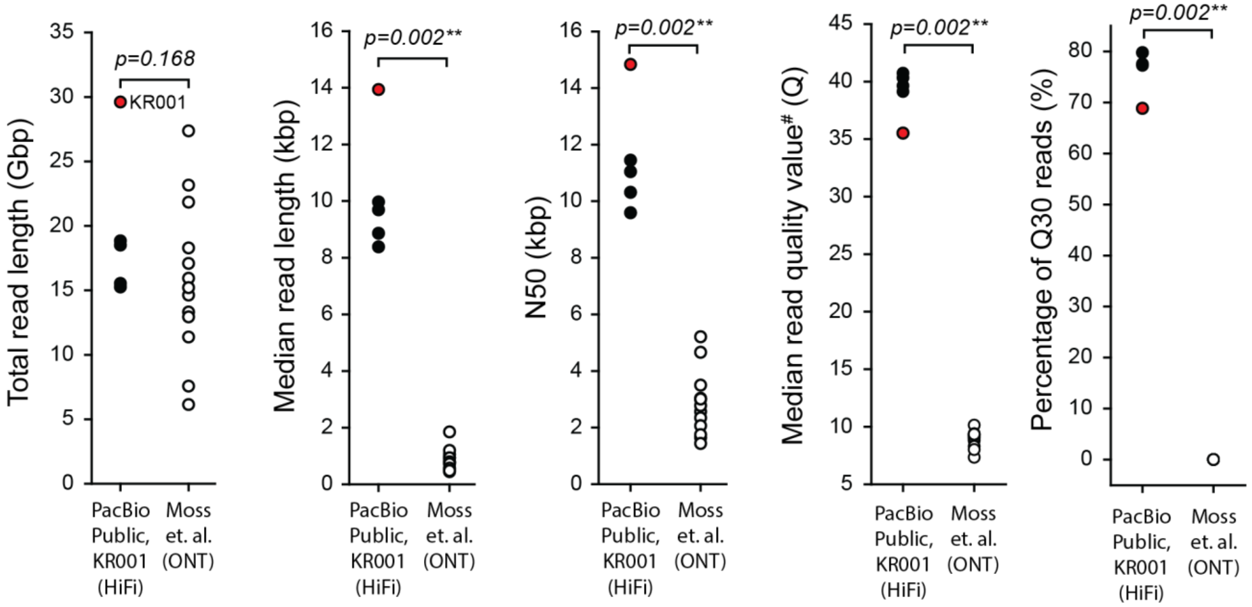

Supplementary Fig. 1 | Comparison of high-accuracy long-read (HiFi) and Oxford nanopore technology (ONT) sequencing data on human feces. Total read length, median read length, N50, median read quality value, and Q30 percentage of HiFi and ONT data were compared using a two-sided Mann–Whitney U test. The red data point represents the in-house HiFi sequencing data from a healthy Korean donor. #Read quality value is $-10\log_{10} e$, where e is expected sequencing error of the read (**$P < 0.01$).

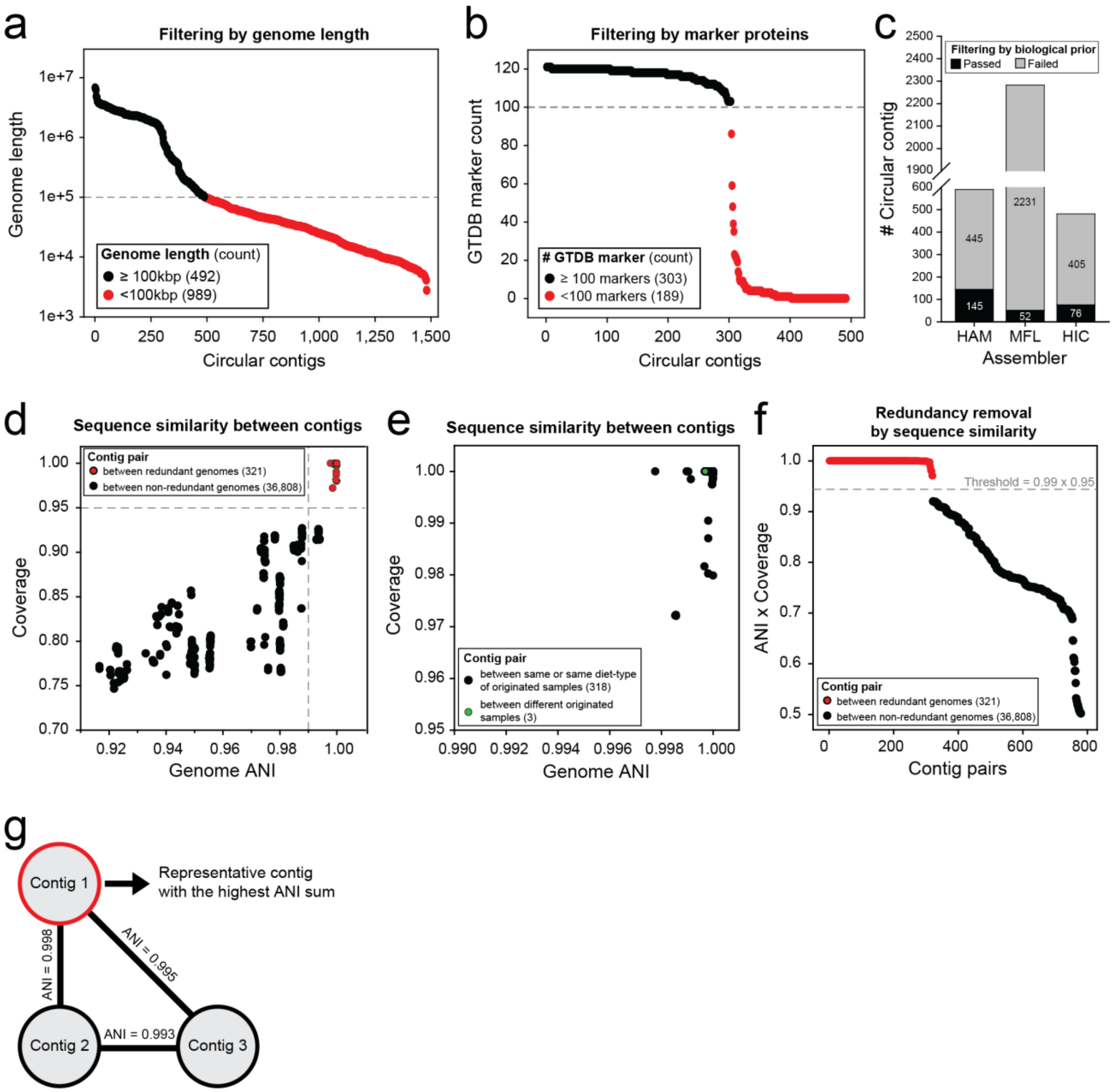

Supplementary Fig. 2 | Filtering of circular contigs by biological priors and removal of redundant contigs. a, b, Filtering of circular contigs by length (a) and number of genome taxonomy database (GTDB) marker proteins (b). Contigs that passed the criteria are denoted in black; whereas those that failed to meet the criteria are denoted in red. **c**, Number of circular contigs before and after the first filtration step for each assembler. **d,** Aaverage nucleotide identity (ANI) and maximum alignment coverage between pairwise circular contigs. Only contig pairs with ANI > 0.9 and maximum alignment coverage > 0.7 are shown in the plot. Contig pairs between redundant contigs with ANI > 0.99 and coverage > 0.95 are highlighted in red. **e,** Highly similar contig pairs with ANI > 0.99 and maximum alignment coverage > 0.95. Most contig pairs were between contigs from the same or the same type of sample (black). **f,** Genome similarity index (ANI × maximum alignment coverage) for contig pairs. The horizontal dashed line represents the score threshold for redundancy removal. Red data points represent contig pairs subjected to redundancy removal. The contig pairs with genome similarity index lower than 0.5 are omitted. **g,** Conceptual diagram showing how the representative contig was selected from the redundant contig group.

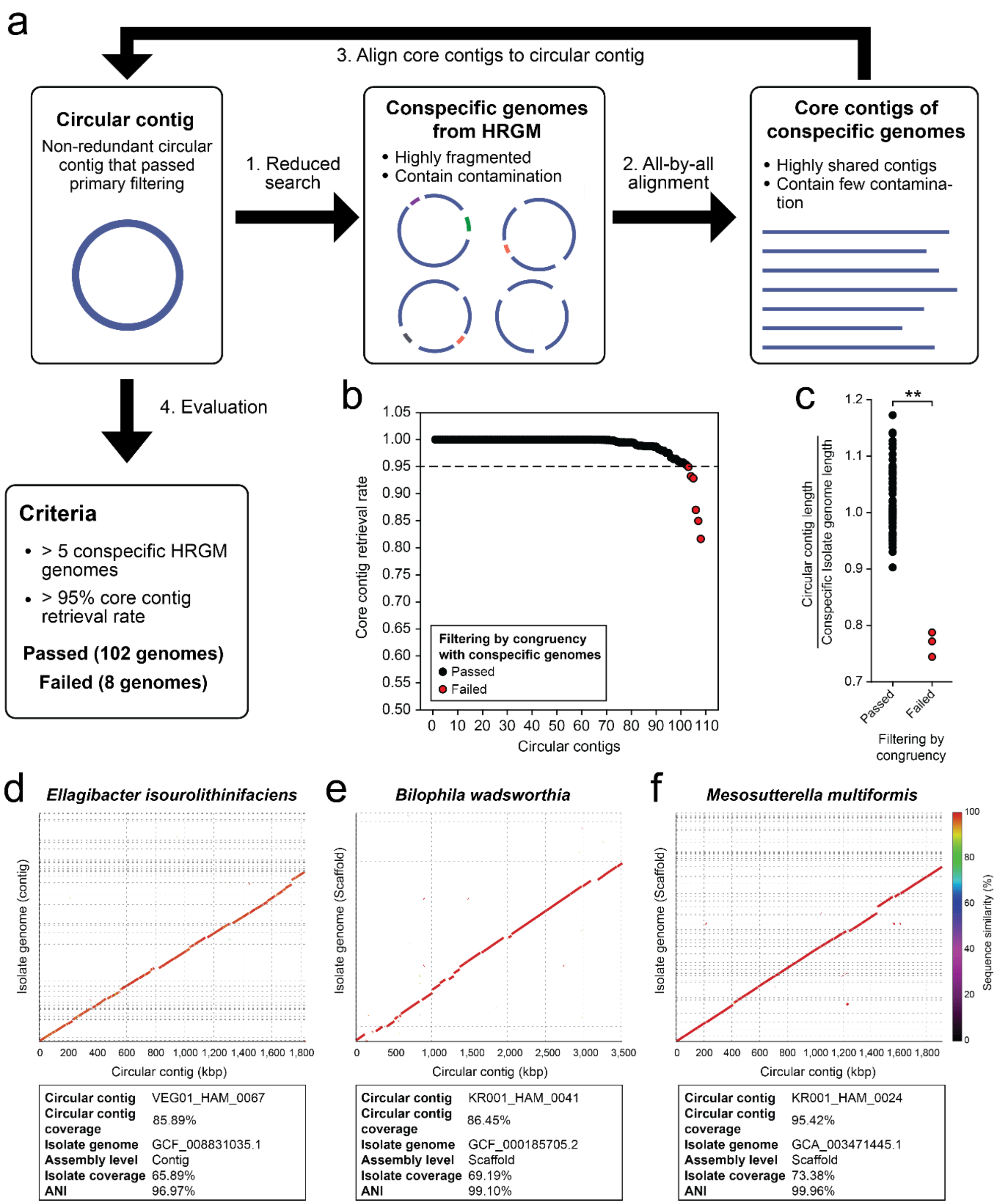

Supplementary Fig. 3 | Filtering circular contigs by congruency with conspecific genomes. a, Schematic diagram illustrating the filtering pipeline. **b,** Filtered circular contigs by core contig retrieval rate. Contigs that passed the filtering threshold are denoted in black; whereas those that failed to pass the threshold are denoted in red. **c,** Relative genome length of circular contigs compared to their conspecific isolated genomes. Relative genome lengths of contigs that passed or did not pass the criteria were compared using the two-sided Mann–Whitney U test. (P-value=0.004) **d–f,** Genome alignment plot between circular contigs that did not pass the criteria and their closest isolated genomes. Genome alignment plot for *Ellagibacter isourolithinifaciens* (d), *Bilophila wadsworthia* (e), and *Mesosutterella multiformis* (f).

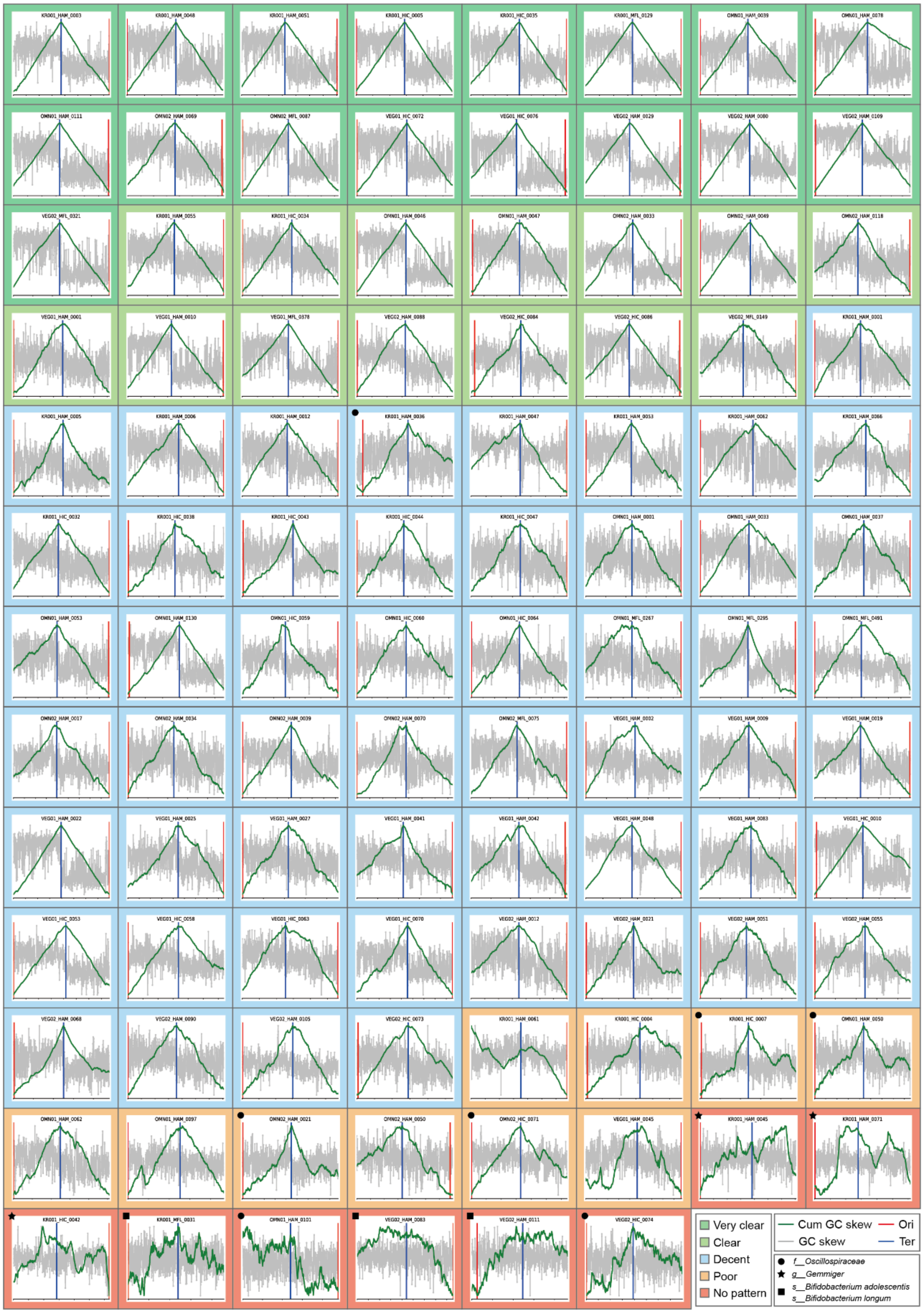

Supplementary Fig. 4 | Five classes of circular contigs based on cumulative GC-skew curves. Different background colors represent different classes. Bacterial lineages repeatedly observed in the poor or no-pattern classes are marked.

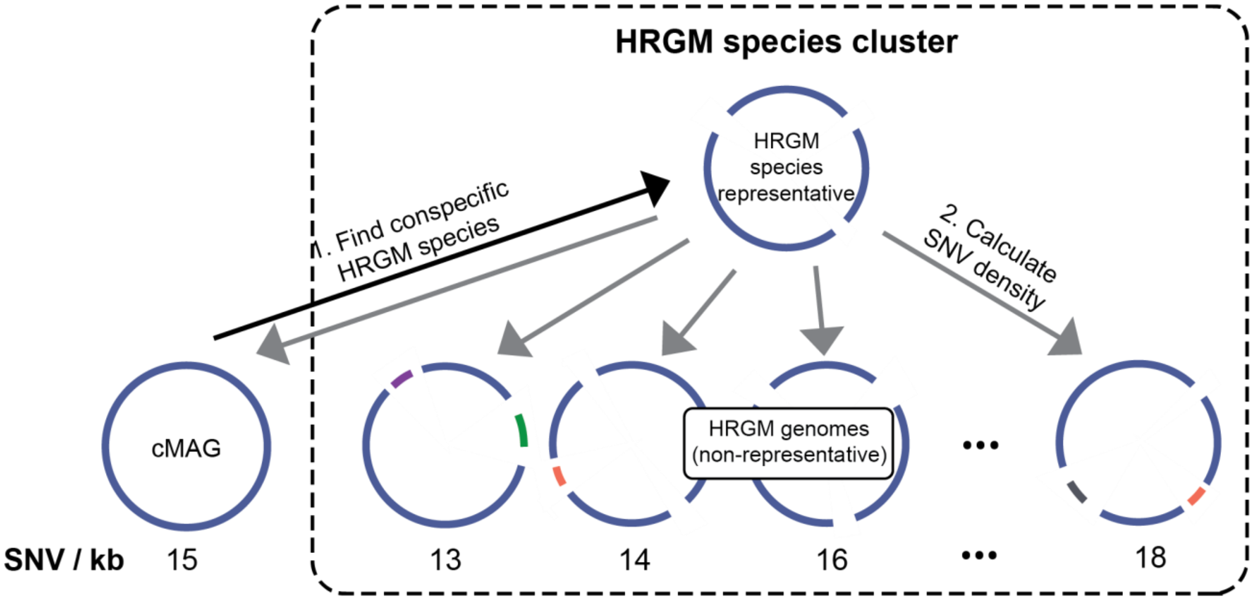

Supplementary Fig. 5 | Schematic diagram representing the pipeline for comparing single nucleotide variation (SNV) density of complete metagenome-assembled genomes (cMAGs) to their conspecific genomes.

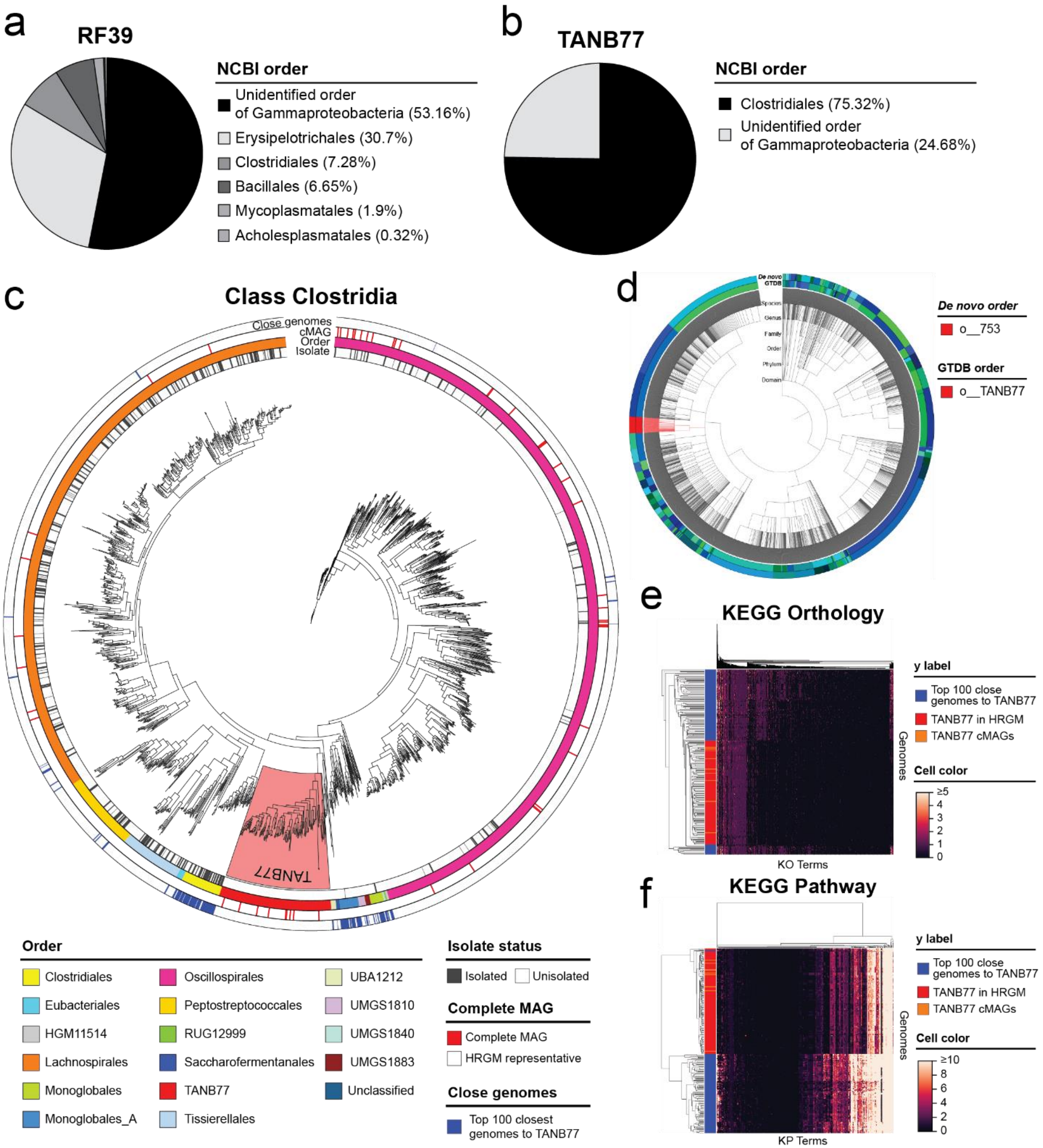

Supplementary Fig. 6 | Complete genomes of entirely uncultured bacterial orders. a, b, Proportion of NCBI orders defining RF39 (a) and TANB77 (b). **c,** Maximum likelihood phylogenetic tree of human reference gut microbiome (HRGM) species and HiFi cMAGs in the Clostridia class. The red-highlighted area represents the TANB77 order. Isolated status, order, HiFi cMAG, and top 100 closest genomes to the TANB77 order are annotated from the innermost to the outermost circle, respectively. **d,** Phylogenetic tree of 4,545 bacterial species of HGM. The inner circle annotates the order according to the GTDB; whereas the outer circle represents the order according to HGM *de novo* classification. TANB77 and o_047 orders are highlighted in red. **e, f,** Hierarchical clustering of heatmaps representing KEGG Orthology (e) and KEGG Pathway (f) profiles of 129 TANB77 (red and orange rows) and the top 100 closest genomes to TANB77 (blue rows).

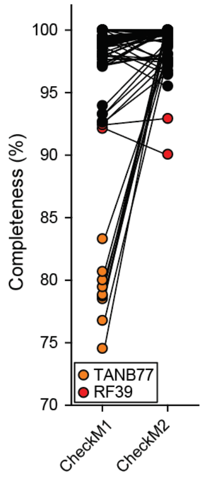

Supplementary Fig. 7 | Completeness of 102 cMAGs assessed by CheckM1 and CheckM2. The completeness scores of the same cMAG (n=102) are connected by a line. Orange and red data points indicate the completeness of TANB77 and RF39 orders, respectively.
