# Supplementary material for: HiFi metagenomic sequencing enables assembly of accurate and complete genomes from human gut microbiota
Source: Nat Commun. 2022 Oct 26;13:6367. doi: 10.1038/s41467-022-34149-0 (PMC9606305; doi:10.1038/s41467-022-34149-0)
Supplement: Supplementary file 3 — Description of Additional Supplementary Files [file 41467_2022_34149_MOESM3_ESM.pdf]

File Name: Supplementary Data 1

Description: Long read metagenomic sequencing samples on human feces

File Name: Supplementary Data 2

Description: List of redundant genomes

File Name: Supplementary Data 3

Description: List of circular contigs by filtering process

File Name: Supplementary Data 4

Description: Final 102 HiFi cMAGs and their information

File Name: Supplementary Data 5

Description: List of taxa without isolated genome

File Name: Supplementary Data 6

Description: List of taxa that are assembled only from KR001 sample

File Name: Supplementary Data 7

Description: Quality assessment of 102 HiFi cMAGs by CheckM v1 and v2
