# Supplementary material for: HiFi metagenomic sequencing enables assembly of accurate and complete genomes from human gut microbiota
Source: Nat Commun. 2022 Oct 26;13:6367. doi: 10.1038/s41467-022-34149-0 (PMC9606305; doi:10.1038/s41467-022-34149-0)
Supplement: Supplementary file 11 — Reporting Summary [file 41467_2022_34149_MOESM11_ESM.pdf]

nature portfolio

Corresponding author(s): Insuk lee

Last updated by author(s): Oct 7, 2022

Reporting Summary

Nature Portfolio wishes to improve the reproducibility of the work that we publish. This form provides structure for consistency and transparency in reporting. For further information on Nature Portfolio policies, see our Editorial Policies and the Editorial Policy Checklist.

Statistics

For all statistical analyses, confirm that the following items are present in the figure legend, table legend, main text, or Methods section.

n/a	Confirmed	
☐	☒	The exact sample size (*n*) for each experimental group/condition, given as a discrete number and unit of measurement
☐	☒	A statement on whether measurements were taken from distinct samples or whether the same sample was measured repeatedly
☐	☒	The statistical test(s) used AND whether they are one- or two-sided<br>*Only common tests should be described solely by name; describe more complex techniques in the Methods section.*
☐	☒	A description of all covariates tested
☐	☒	A description of any assumptions or corrections, such as tests of normality and adjustment for multiple comparisons
☐	☒	A full description of the statistical parameters including central tendency (e.g. means) or other basic estimates (e.g. regression coefficient) AND variation (e.g. standard deviation) or associated estimates of uncertainty (e.g. confidence intervals)
☐	☒	For null hypothesis testing, the test statistic (e.g. *F*, *t*, *r*) with confidence intervals, effect sizes, degrees of freedom and *P* value noted<br>*Give P values as exact values whenever suitable.*
☒	☐	For Bayesian analysis, information on the choice of priors and Markov chain Monte Carlo settings
☒	☐	For hierarchical and complex designs, identification of the appropriate level for tests and full reporting of outcomes
☒	☐	Estimates of effect sizes (e.g. Cohen's *d*, Pearson's *r*), indicating how they were calculated

Our web collection on statistics for biologists contains articles on many of the points above.

Software and code

Policy information about availability of computer code

Data collection	No software was used for the data collection.

Data analysis

minimap2 (v2.18-r1015)
Canu (v2.1.1)
metaFlye (v2.8.3-b1695)
hifiasm_meta (v0.2-r040)
GTDB-Tk (v1.6.0, db: r202)
CheckM (v1.1.3)
Barrnap (v0.9)
tRNAScan-SE (v2.0.7)
nucmer (v4.0.0beta2)
EggNOG-mapper (v2.1.6, db: eggNOG 5.0)
Prokka (v1.14.6)
IslandViewer4 (v4, web software: https://www.pathogenomics.sfu.ca/islandviewer/)
IQ-Tree (v2.1.3)
ITOL (v6, web software: https://itol.embl.de/)
Bubblegun (v1.1.1)
Codes developed for this study (cMAGfilter) is deposited at github (https://github.com/netbiolab/cMAGfilter)

For manuscripts utilizing custom algorithms or software that are central to the research but not yet described in published literature, software must be made available to editors and reviewers. We strongly encourage code deposition in a community repository (e.g. GitHub). See the Nature Portfolio guidelines for submitting code & software for further information.

Data

Policy information about availability of data

All manuscripts must include a data availability statement. This statement should provide the following information, where applicable:

- Accession codes, unique identifiers, or web links for publicly available datasets
- A description of any restrictions on data availability
- For clinical datasets or third party data, please ensure that the statement adheres to our policy

The public HiFi metagenomic sequencing data for pooled human fecal samples are available from NCBI Sequence Read Archive (PRJNA750084). HiFi metagenomic sequencing data for a Korean fecal sample generated in this study are deposited in the Sequence Read Archive (PRJNA798244). The entire 102 cMAGs sequences and their GC-skew and genome bin retrieval rate plots are available at https://doi.org/10.5281/zenodo.5996768. The short-read sequencing based human gut microbiome genome catalog is available from the HRGM database (https://www.mbiomenet.org/HRGM/). The additional list of cultured genomes is obtained from the hGMB database (https://hgmb.nmdc.cn/). Human reference genome is downloaded from NCBI-Assembly (https://www.ncbi.nlm.nih.gov/assembly/GCF_000001405.39/).

Human research participants

Policy information about studies involving human research participants and Sex and Gender in Research.

Reporting on sex and gender	Fecal sample from one Korean male volunteer.
Population characteristics	Sex: Male<br>Nationality: Korean<br>Age: 55<br>(We did not made any conclusion with the characteristics of the sample)
Recruitment	We publicly recruited the fecal sample donor from Yonsei University and selected one healthy Korean volunteer. As the study is not for comparison there is no potential bias that could affect the conclusion of the study. The volunteer was informed about the sampling process and the purpose of the research, and fully consented.
Ethics oversight	Yonsei University Institutional Review Board approved the study (IRB No. 4-2020-0309).

Note that full information on the approval of the study protocol must also be provided in the manuscript.

Field-specific reporting

Please select the one below that is the best fit for your research. If you are not sure, read the appropriate sections before making your selection.

[x] Life sciences [ ] Behavioural & social sciences [ ] Ecological, evolutionary & environmental sciences

For a reference copy of the document with all sections, see nature.com/documents/nr-reporting-summary-flat.pdf

Life sciences study design

All studies must disclose on these points even when the disclosure is negative.

Sample size	One fecal sample from a healthy Korean volunteer was used in this study. As the study is not for comparison, large sample size was not required. To obtain higher-quality genome we decided to increase sequencing depth rather than increase sample size.
Data exclusions	There was no data exclusion.
Replication	No measurement that requires replication was performed.
Randomization	The fecal sample was used for long-read sequencing followed by cMAG assembly. The study does not include any conclusion that require randomization.
Blinding	The fecal sample was used for long-read sequencing followed by cMAG assembly. The study does no contain any results that can be affected by blinding.

Reporting for specific materials, systems and methods

We require information from authors about some types of materials, experimental systems and methods used in many studies. Here, indicate whether each material, system or method listed is relevant to your study. If you are not sure if a list item applies to your research, read the appropriate section before selecting a response.

Materials & experimental systems

n/a	Involved in the study	
☒	☐	Antibodies
☒	☐	Eukaryotic cell lines
☒	☐	Palaeontology and archaeology
☒	☐	Animals and other organisms
☒	☐	Clinical data
☒	☐	Dual use research of concern

Methods

n/a	Involved in the study	
☒	☐	ChIP-seq
☒	☐	Flow cytometry
☒	☐	MRI-based neuroimaging
